# APT Weighted MRI as an Effective Imaging Protocol to Predict Clinical Outcome After Acute Ischemic Stroke

**DOI:** 10.3389/fneur.2018.00901

**Published:** 2018-10-23

**Authors:** Guisen Lin, Caiyu Zhuang, Zhiwei Shen, Gang Xiao, Yanzi Chen, Yuanyu Shen, Xiaodan Zong, Renhua Wu

**Affiliations:** ^1^Department of Medical Imaging, The Second Affiliated Hospital, Medical College of Shantou University, Shantou, China; ^2^Department of Mathematics and Statistics, Hanshan Normal University, Chaozhou, China; ^3^Provincial Key Laboratory of Medical Molecular Imaging, Shantou, China

**Keywords:** APT, CEST, stroke, prognosis, magnetic resonance imaging

## Abstract

To explore the capability of the amide-proton-transfer weighted (APTW) magnetic resonance imaging (MRI) in the evaluation of clinical neurological deficit at the time of hospitalization and assessment of long-term daily functional outcome for patients with acute ischemic stroke (AIS). We recruited 55 AIS patients with brain MRI acquired within 24–48 h of symptom onset and followed up with their 90-day modified Rankin Scale (mRS) score. APT weighted MRI was performed for all the study subjects to measure APTW signal quantitatively in the acute ischemic area (APTW_ipsi_) and the contralateral side (APTW_cont_). Change of the APT signal between the acute ischemic region and the contralateral side (ΔAPTW) was calculated. Maximum APTW signal (APTW_max_) and minimal APTW signal (APTW_min_) were also acquired to demonstrate APTW signals heterogeneity (APTW_max−min_). In addition, all the patients were divided into 2 groups according to their 90-day mRS score (good prognosis group with mRS score <2 and poor prognosis group with mRS score ≥2). In the meantime, ΔAPTW of these groups was compared. We found that ΔAPTW was in good correlation with National Institutes of Health Stroke Scale (NIHSS) score (*R*^2^ = 0.578, *p* < 0.001) and 90-day mRS score (*R*^2^ = 0.55, *p* < 0.001). There was significant difference of ΔAPTW between patients with good prognosis and patients with poor prognosis. Plus, APTW_max−min_ was significantly different between two groups. These results suggested that APT weighted MRI could be used as an effective tool to assess the stroke severity and prognosis for patients with AIS, with APTW signal heterogeneity as a possible biomarker.

## Introduction

As a promising contrast mechanism, chemical exchange saturation transfer (CEST) has become an important tool in the field of molecular imaging (1). Recently, APTW MRI, one form of CEST technology, has been increasingly applied in capturing tissue acidosis as a research tool based on its capability to detect pH and mobile proteins content (2). APTW MRI has been used to assess the severity of tissue acidification in hyperacute and acute stroke (3, 4). For all these pre-clinical researches, the induced stroke studies were carried out under highly controlled environment and the animals were scanned during early stage of stroke within hours. Under this circumstance, the APTW imaging was called pH-weighted imaging since pH was the major factor to affect the APTW signal intensity, accounting for more than 90% (5). Clinical assessment using APTW imaging is considered promising given its ability to characterize pH of the stroke area within hours from the symptom onset. The enthusiasm of applying the APTW imaging to patients with none hyperacute stroke (within hours from symptom onset) might be decreased given the fact that many factors can affect the APTW signal (5). However, a considerable proportion of patients with stroke have delay in presentation to the hospital (6, 7). Applying of APTW imaging might be clinically useful given the large number of patients with relatively delayed presentation to the hospital. In this study, we would like to testify the capability of APTW MRI as a tool to assess stroke severity as well as to predict clinical outcome of patient of acute ischemic stroke (AIS) with symptom onset between 24 and 48 h by measuring the change of APTW signal intensity.

## Phantoms, patients, and methods

### Phantoms

Bull serum albumin (BSA) was used to optimize and characterize the APT signal as well as to confirm the pH dependent APT CEST effect (8). Four cylinders of 20% BSA solution with different pH values (6.0, 6.4, 6.8, 7.2) were prepared, which were bundled together.

### Participants

This study was approved by the local ethics committee of the Second Affiliated Hospital of Shantou University Medical College. Informed written consent was obtained from participants or legal guardians when patients were unable to provide consent before the study. From September 2016 to January 2018, all participants (>18 years old) presenting to the Second Affiliated Hospital of Shantou University Medical College with clinical signs and symptoms of AIS confirmed by diffusion weighted imaging (DWI) performed within 24–48 h of symptoms onset, were eligible for enrollment in this study. During this time interval, the stroke was defined as acute stroke according to Huang et al's study (9) and Baird et al's study (10). Exclusion criteria included receiving intravenous t-PA treatment before APT MRI scan, a history of known other neurologic disorders (brain tumor, brain trauma, etc.), insufficient image quality caused by motion artifacts, very small acute ischemic lesion, a confirmed diagnosis of stroke caused by a systemic illness (bacterial endocarditis, vasculitis, etc.), and brainstem stroke. We included 55 participants (36 men and 19 women, age range from 20 to 87).

### Clinical assessment and outcome measure

All patients were evaluated by at least a neurologist when they were admitted to the hospital with NIHSS score to assess their clinical stroke severity. A 90-day mRS score was obtained by a well-trained staff through telephone interview of the patients or their legal guardians to assess their long-term clinical outcome.

### MR imaging techniques and scanning process

All MR images were acquired on a 3.0-T MRI scanner (Sigma; GE Healthcare, Milwaukee, WI, U.S.), using an 8-channel phased-array head coil. Sponge padding was used to limit head motion. T2-weighted images (T2WI) [repetition time (TR) = 4,480 ms, echo time (TE) = 120 ms], T2WI fluid attenuated inversion recovery images (TR = 8,600 ms, TE = 155 ms, Inversion Time = 2,100 ms), and diffusion-weighted images (TR = 6,000 ms; TE = minimum; b-value = 1,000 s/mm^2^) were performed to acquire information on the brain of all subjects.

The CEST scan was based on a MT-prepared Echo-planar imaging (EPI) MRI sequence. The bovine serum albumin (BSA) phantoms were used to optimize the APT sequence. All the parameters included TR, TE, bandwidth, number of saturation pulse were firstly adjusted. Then different flip angles (100°, 180°, 260°, 340°) were used to generate different RF irradiation power to obtain clear APTW imaging contrast. The duty cycle was fixed to 50%. For all the patients, the following optimized setting was used: TR = 5,000 ms, TE = 3.1 ms, FOV = 240 × 240 mm^2^, matrix = 128 × 128, 1 slice, slice thickness = 5 mm, bandwidth = 15.63 kHz. The MT saturation pulse was a Fermi pulse with a 40 ms width, a flip of 340°, duty cycle of 50% and the number of saturation pulse of 50 for an RF irradiation power of 1 μT. Forty-one equidistant frequency offsets in the range of 5 to −5 ppm and an additional S0 image without irradiation were acquired. B0 inhomogeneity was corrected using a water saturation shift referencing map (11). The average measurement time for CEST in this study is around 30 min. To limit the effect of motion artifact, the whole scanning process was as follows. We obtained one imaging with a certain frequency offset and then changed the offset to get another imaging. The operator could observe whether the patient move significantly or not during the scanning. Once significant movement was observed, the scanning would be paused. Re-scanning would be started after the patient was repositioned and re-instruction was given to the patient. Extremely irritable patients were not recruited in our study, which was suggested by the neurologists in our hospital. Even with all the methods we described above, we did find 5 patients with significant movement during data analysis that could not be used for analysis. These 5 patients were excluded. For those with mild movement that just had mid rotation of the head which did not change the plane we chose, correction using SPM software was available in our laboratory.

### Data processing and analysis

We used Matlab 7 (Mathworks, Natick MA, U.S.) to process the APT MRI data. The APT map was calculated by using the equation:

$$\begin{array}{l} {\text{MTR}}_{\text{asym}}\left(3.5\text{ ppm}\right)=\frac{\text{S}\left(-3.5\text{ ppm}\right)-\text{S}\left(+3.5\text{ ppm}\right)}{{\text{S}}_{0}} \end{array}$$

**Figure 1:**
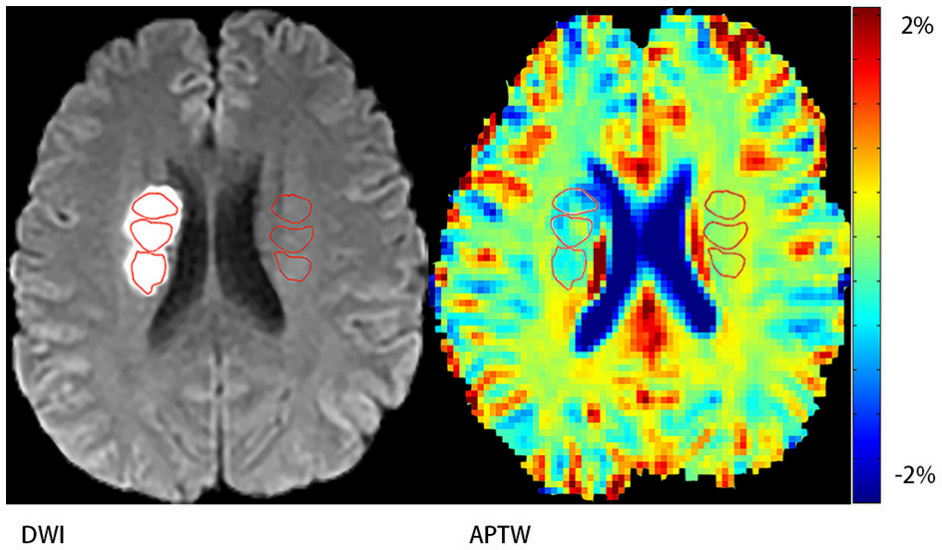
Demonstration of regions of interest (ROIs). Three ROIs on the stroke hemisphere within the stroke area defined by DWI and another three ones on the contralateral hemisphere.

In this study we referred MTR_asym_ (3.5 ppm) as APTW signal intensity. Three different regions of interest (ROIs) that did not overlap were chosen in the acute ischemic area defined by DWI image and another 3 different ROIs in the contralateral side (Figure 1). Then the average APTW signal intensity of the acute ischemic area (APTW_ipsi_) and the contralateral side (APTW_cont_) were calculated. Change of the APTW signal intensity between the acute ischemic region and the contralateral side (ΔAPTW) was calculated. In the case of bilateral ischemic stroke, which happened in 2 of our patients, we chose 3 different ROIs in the surrounding normal appearing region. For convenience, we also referred this as APTW_cont_. Plus, the maximal APTW signal intensity (APTW_max_) and the minimal APTW signal intensity (APTW_min_) of the acute ischemic area in the same patient were reported and the difference between them (APTW_max−min_) that reflected APTW signal heterogeneity was calculated. The APTW_max_ and APTW_min_ were defined as follows. Thirty small ROIs with each covered 4 voxels were placed evenly in the stroke area. The upper quartile of the values was defined as APTW_max_ whereas the lower quartile of the values was defined as APTW_min_. The measurement was carried out by a staff that had been trained to use Matlab 7 and was blinded to the NIHSS score as well as mRS score of the patients.

The infarct volume of the acute stroke area was measured using MRIcro (University of Nottingham School of Psychology, Nottingham, UK; www.mricro.com). The images were converted from DICOM into HDR for MRIcro analysis. The acute infarct region was delineated manually on the T2FLAIR sequence since the DWI sequence for our patients did not cover the full brain.

Pearson's correlation test was used to measure the relationship between the ΔAPTW and the NIHSS score and mRS score separately. Two-sample *t*-test was used to compare the difference between the high NIHSS group and low NIHSS group. Data analysis was performed using statistical package SPSS16.0 for Windows. A significant level was set at the level of *p* < 0.05.

## Results

### Phantoms

**Figure 2:**
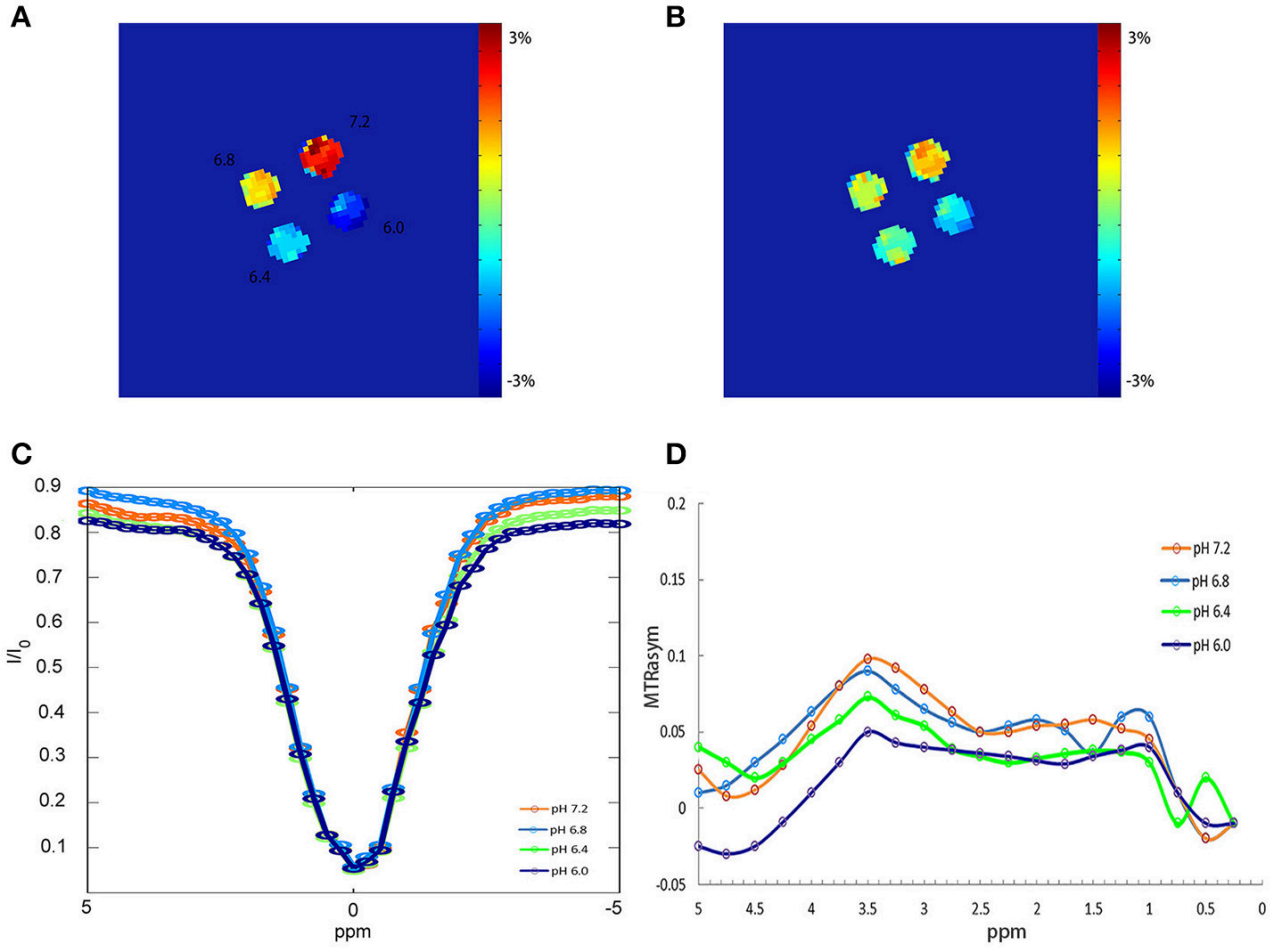
APTW imaging, Z spectra and MTRasym curves of BSA phantoms. **(A)** APTW imaging of 4 20% BSA phantoms with difference pH (6.0, 6.4, 6.8, 7.2) measured at a width of 40 ms, a flip of 340°, duty cycle of 50% and the number of saturation pulse of 50. **(B)** APTW imaging of 4 different pH BSA phantoms measured at a width of 40 ms, a flip of 180°, duty cycle of 50%, and the number of saturation pulse of 50. **(C,D)** Z spectra and MTRasym curves of 4 different pH BSA phantom measured with a flip of 340°.

Figure 2 shows APTW imaging, Z spectra and MTR_asym_ curves of BSA solutions. APTW imaging of 4 BSA solutions (20%) with different pH values (6.0, 6.4, 6.8, 7.2) could be seen from (A) to (B). Four phantoms showed clear and good contrast, which was consistent with the previous study (12). (A) Imaging was acquired with a flip angle of 340°, while (B) was acquired with a flip angle of 180° (all other parameters were the same as the ones described above). We can tell that with a flip angle of 340°, the phantom showed more clear contrast. Both the Z spectra (C) and the MTR_asym_ curves (D) of the BSA solutions with different pH acquired with a flip angle of 340°showed noticeable asymmetry around 3.5 ppm for 4 different pH BSA solutions.

### Clinical characteristics of 55 patients with AIS

We initially recruited 63 patients and 55 patients remained in our study. For the patients who were excluded, 2 had very small acute ischemic area, 5 had significant motion artifact, and 1 had received t-PA treatment before the MR scanning. For all these patients that remained in this study (mean age was 66.1 ± 13.1 years), 38 had hypertension (69.1%), 10 had coronary artery disease (18.2%), 25 had diabetes mellitus (45.5%), 6 had atrial fibrillation, 16 had hyperlipidemia, and 25 had a long history of smoking (45.5%). The median NIHSS score was 4. For the 90-day mRS score, 33 had a score lower than or equal to 1 and 22 had a score higher than or equal to 2 with 1 of them died within 90 days. The basic clinical characteristics of the patients were listed in Table 1.

**Table 1:** Baseline clinical characteristics of 55 patients with AIS (*n* = 55).

Mean age (SD), y	66.1 (13.1)
Male, *n* (%)	36 (65.5)
**Medical history, *n* (%)**	
Hypertension	38 (69.1)
Coronary artery disease	10 (18.2)
Diabetes mellitus	25 (45.5)
Atrial fibrillation	6 (10.9)
Hyperlipidemia	16 (29.1)
Smoking	25 (45.5)
**Admission data**	
Systolic blood pressure, Mean (SD), mm Hg	162.4 ± 24.0
Diastolic blood pressure, Mean (SD), mm Hg	95.0 ± 20.8
NIHSS score, median	4
**Follow-up mRS score, *n* (%)**	
0	12 (21.8)
1	21 (38.2)
2	10 (18.2)
3	6 (10.9)
4	3 (5.5)
5	2 (3.6)
6	1 (1.8)

*AIS, acute ischemic stroke; NIHSS, NIH Stroke Scale; mRS, modified Rankin Scale*.

### APTW images of stoke patients

**Figure 3:**
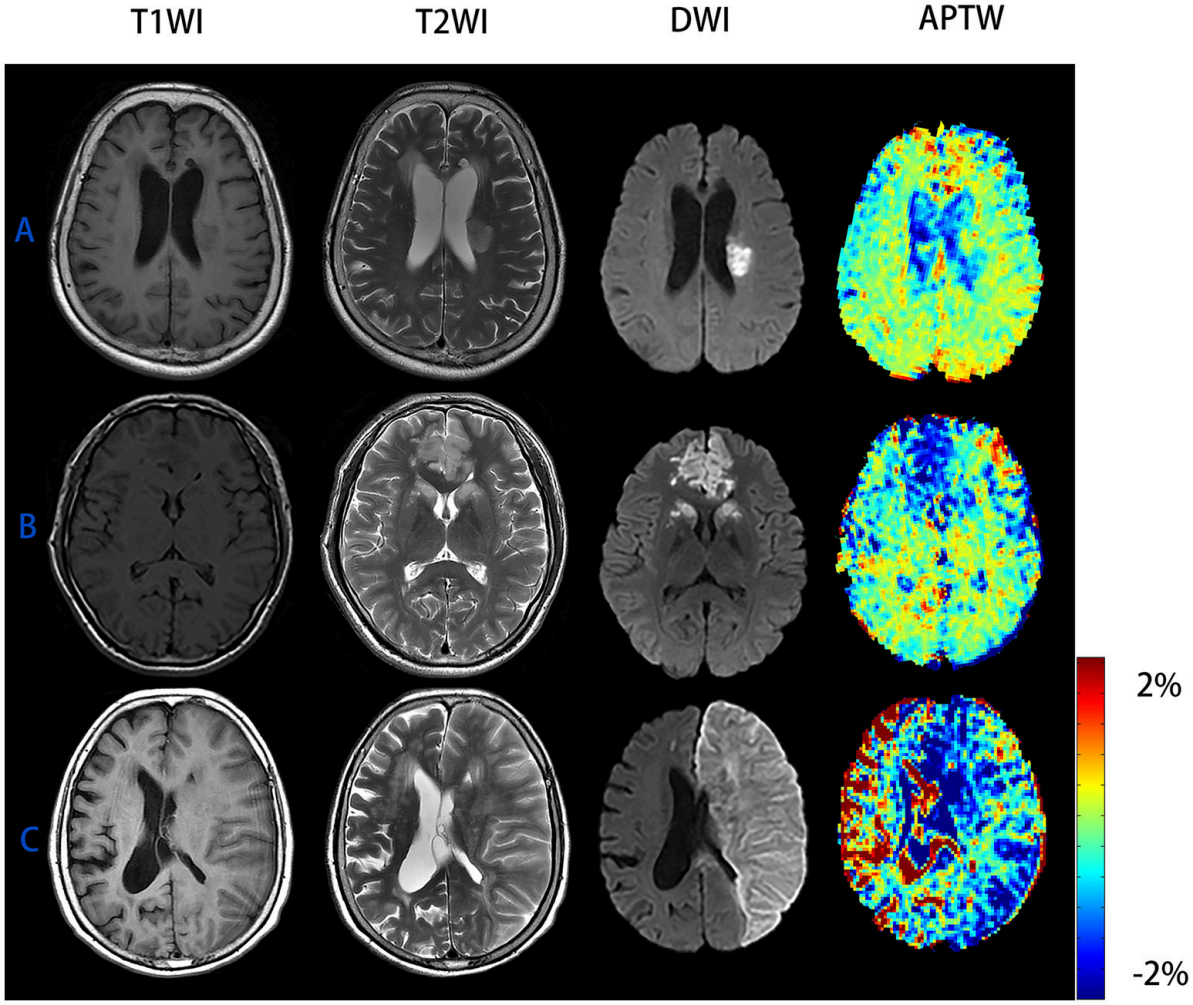
Conventional MR images and APTW images of patient with AIS of different NIHSS scores and mRS scores. **(A)** M/65 years, NIHSS score was 3 and 90-day mRS score was 0, ΔAPTW = −0.37%. **(B)** F/69 years, NIHSS score was 5 and 90-day mRS score was 2, ΔAPTW = 0.82%. **(C)** M/81 years, NIHSS score was 22 and 90-day mRS score was 6, ΔAPTW = 1.93%. The criterion for patient with good prognosis (mRS score <2) is ΔAPTW > −0.783.

We can see from the image (Figure 3) that patient C had the highest mRS score and NIHSS score among patient A to C but his APTW signal was the lowest one. While comparing patient A to C, APTW signal intensities in the ischemic region decreased and the signal intensities were visibly lower in patients with higher NIHSS scores and mRS scores. What's more, APT signal heterogeneity was more obvious for patient C than patient A and B. The lesion size showed in the APTW imaging was approximately equal to that identified by conventional DWI.

### Correlation between ΔAPTW signal and NIHSS score/mRS score, ROC analysis for ΔAPTW to predict good/poor outcome

**Figure 4:**
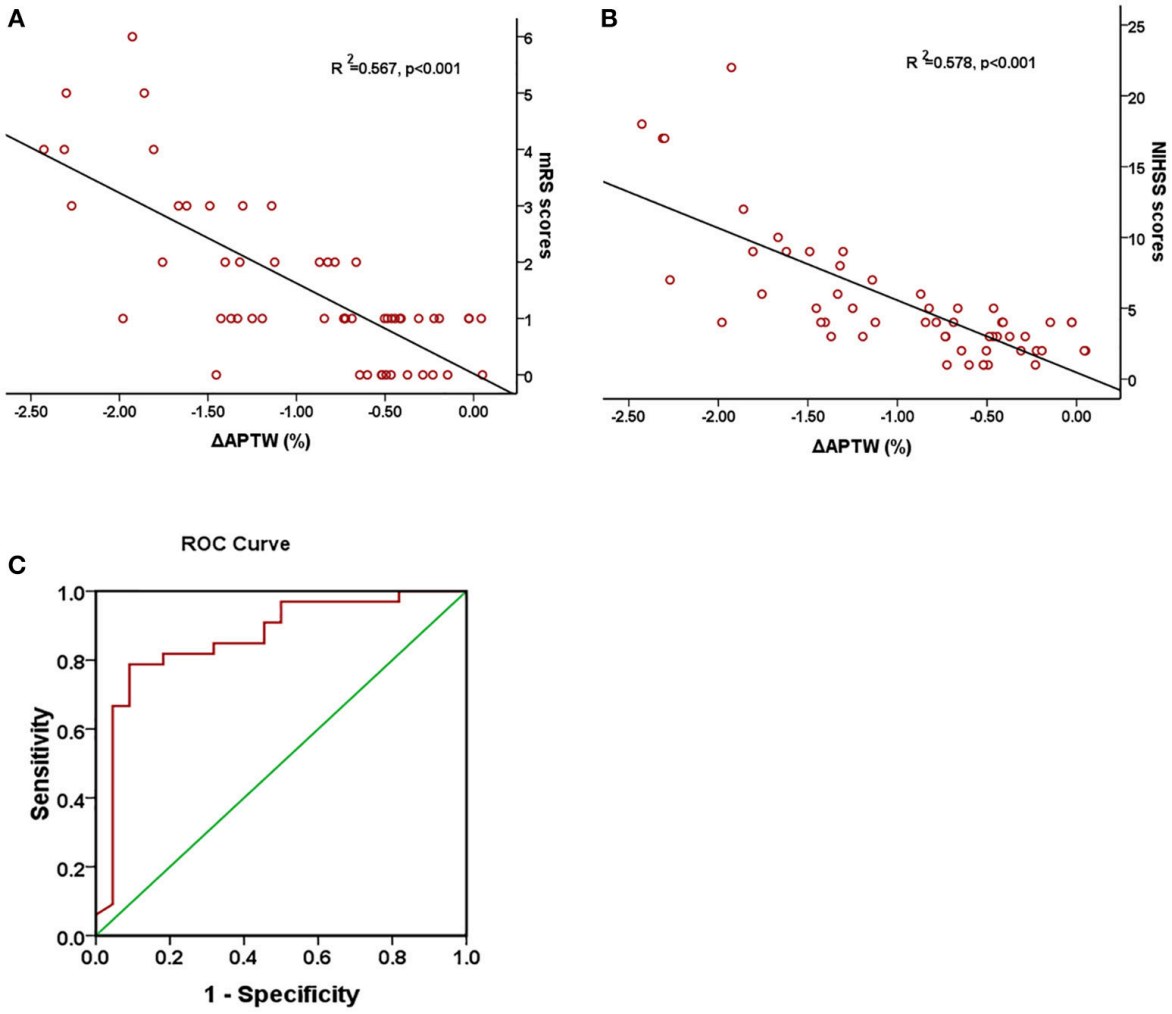
Association between ΔAPTW and NIHSS score and 90-day mRS score. Pearson regression test showed a good correlation between 90-day mRS score **(A)** and NIHSS score **(B)** with ΔAPTW. Receiver operating characteristic (ROC) curves of ΔAPTW to predict good/poor outcome **(C)**. The area under the curve is 0.864.

Figure 4 shows Pearson correlation test of the relationship between the change of APTW signal intensity (ΔAPTW) and the NIHSS score, 90-day mRS score, respectively as well as ROC curve forΔAPTW to predict good/poor outcome. As it could be seen, the APTW signal intensity decreased more as the NIHSS score and the mRS score went higher. The ΔAPTW signal showed good correlation with both the NIHSS (*R*^2^ = 0.578, *p* < 0.001) and mRS score (*R*^2^ = 0.55, *p* < 0.001). However, for patients with same NIHSS or same mRS score, ΔAPTW varied to certain extend. The area under the curve is 0.864, which means the ΔAPTW has a relatively good capacity to predict patient outcome. The criterion for good outcome is ΔAPTW > −0.783. The sensitivity and the specificity of this criterion to predict good outcome are 78.8 and 90.9%, respectively.

### Baseline characteristics of 2 groups of patients with different clinical outcome

Table 2 shows the baseline clinical characteristics and multiple APT signal intensity parameters of patients in two different groups divided according to their 90-day mRS score. Patients in both groups had similar baseline clinical characteristic except for NIHSS score and the infarct volume (both *p* < 0.05). In addition, both APTW_ipsi_ and ΔAPTW were significantly lower in the poor prognosis group than the good prognosis group (*p* < 0.001). However, there was no significant difference of APTW_cont_ between two groups. As for APTW_max−min_, poor prognosis group had significant higher value than good prognosis group (*p* = 0.044).

**Table 2:** Baseline characteristic and multiple APT signal parameters of two groups AIS patients.

	**Mean** ±**SD or** ***n*** **(%) or median (IQR)**		***p*-value**
	**AIS patients with good prognosis (*n* = 33)**	**AIS patients with poor prognosis (*n* = 22)**
Sex, male	22 (66.7)	14 (63.6)	0.817
Age, y	65.2 ± 11.9	67.5 ± 14.9	0.516
Hypertension	23 (69.7)	15 (68.2)	0.905
Diabetes mellitus	15 (45.5)	10 (45.5)	1.000
Atrial fibrillation	2 (6.1)	3 (13.6)	0.338
Total cholesterol, mmol/L	5.3 ± 1.4	5.3 ± 1.5	0.874
Triglyceride, mmol/L	1.6 ± 1.3	1.6 ± 1.5	0.963
HDL cholesterol, mmol/L	1.3 ± 0.3	1.2 ± 0.4	0.530
HbA1c, %	7.2 ± 2.9	7.5 ± 4.0	0.691
LDL cholesterol, mmol/L	3.0 ± 1.1	3.2 ± 1.2	0.497
Smoking	15 (45.5)	10 (45.5)	1.000
NIHSS score	3 (2–4)	7.5 (5–10.5)	< 0.001
Infarct volume (cm^3^)	1.96 (0.98–4.52)	14.12 (8.74–25.56)	0.039
APTW_ipsi_ (%)	−1.7 ± 0.6	−2.6 ± 0.8	< 0.001
APTW_cont_ (%)	−1.4 ± 1.8	−1.1 ± 0.6	0.468
ΔAPTW (%)	−0.6 ± 0.5	−1.5 ± 0.7	< 0.001
APTW_max−min_ (%)	0.7 ± 0.3	1.0 ± 0.2	< 0.001

*AIS, acute ischemic stroke; HDL, high density lipoprotein; LDL, low density lipoprotein*.

## Discussion

In this study, we used APTW MRI to investigate the relationship between the change of the APTW signal intensity in AIS patients and their NIHSS score as well as their 90-day mRS score. The results of this analysis demonstrated that the change of the APT signal intensity had good correlation (*R*^2^ = 0.578, *p* < 0.001) with the NIHSS score as well as the 90-day mRS score (*R*^2^ = 0.55, *p* < 0.001), which indicated that it could be used as a tool to access stroke severity and predict stroke outcome. Previous studies have shown that APT MRI is capable of detecting tissue pH (13–16). Moreover, researches performed in animal models with ischemic stroke have indicated that amide proton transfer ratio, which was calculated as MTR_asym_ (3.5 ppm), was in good correlation with pH and the concentration of lactic acid (14, 17). Notably, all these experiments were carried out very soon after the stroke occurred (within several hours) in animal models with highly controlled conditions. In the time interval of our patients, it was still not sure what percentage pH had contributed to the change of the APTW signal given the fact that many factors could affect the APTW signal intensity. Nevertheless, tissue acidification and significantly increased lactate level still exist during this time interval, which has been proven previously (18). And further study with more precise control should be conducted. Obviously, the prognosis could be predicted by NIHSS score or the volume of the acute lesion. But the use of APTW MRI may reveal tissue microenvironment, including tissue acidification and protein concentration, which provides insight for better understanding the pathophysiological process of acute stroke lesion in addition to predict the outcome.

Even though ΔAPTW showed linear correlation with NHISS score as well as mRS score, we did find that for patients with the same NIHSS score or mRS score, the ΔAPTW could be varied to a relatively large extend (e.g., for patients with 90-day mRS score of 0, the maximum ΔAPTW was 0.5% and the minimum ΔAPTW was −1.5%.). This was shown by the *R*^2^, which indicated only moderate goodness-of-fit. To explore whether patients of same NIHSS score but lower ΔAPTW would have poorer prognosis, we subsequently divided patients with NIHSS score of 4 (12 patients) into 2 groups according to their ΔAPTW. As we learn from the ROC analysis, ΔAPTW ≤ −0.783 means poor prognosis and ΔAPTW > −0.783 means good prognosis. We found that in 6 patients with ΔAPTW > −0.783, 5 of them had mRS score <2 and 1 of them had mRS score ≥2. For the other 6 patients with ΔAPTW ≤ −0.783, 3 of them had mRS score ≥2 and 3 of them had mRS score <2. However, the result was not significant (*p* > 0.05, Chi-square test) due to small sample size and mixed results. It is known that as one inherent problem of the NIHSS scale, certain categories on the scale that earn one point do not impair the overall life quality of the patient (like facial paresis) whereas other categories will both earn one point on the NIHSS scale and lead to a lower mRS score (like leg paralysis). This explained why the prognosis of patients with same NIHSS score varied (as showed by mRS scores). And it may partially explain why patients with same NIHSS had various ΔAPTW signals if ΔAPTW could be used to predict stroke outcome. But this seems no to be the whole picture. We believe that several factors besides pH that affect that APTW signal, such as tissue temperature (2), amine protein concentration (19), T1 (17), edema and possibly spontaneous reperfusion at the time of imaging, would possibly lead to various ΔAPTW measurements for patients with same NIHSS score or same mRS score.

In addition, we divided the patients into 2 groups according to their mRS scores. For these two groups of patients, no significant difference had been found in their baseline clinical risk factor (all *p* > 0.05). Plus, no difference was found between the APTW signal intensity of the non-lesion side in two groups, which was consistent with Song's study (16). Not surprisingly, both the NIHSS score and infarct volume of two groups showed significant difference. It had been reported that infarct volume was a good prognostic factor for stroke patients (20–22). Furthermore, we found that there had no difference between the APTW_ipsi_ and APTW_cont_ in the group with low mRS score, whereas patients in the other group showed significant difference between the APTW_ipsi_ and APTW_cont_. The possible explanation is that the change of the affecting factors (pH, protein concentration, etc.) in the brain tissue of patients with mild neurological deficit is relatively slight that they could possibly return to nearly normal, so that the APT MRI could not detect in the time interval of 24–48 h after stroke onset for patients with mild symptoms.

For the heterogeneity of APTW signals in the stroke area showed by APTW_max−min_, we found that patients in the poor prognosis group had more heterogeneous APTW signals than patients in the good prognosis (*p* < 0.001). Previous studies have noticed the variety of APT signal intensity that exist between the oligemia area and the ischemic core which suggested pH differed in the stroke area defined by traditional diffusion weighted images (23). One possibility was that pH inhomogeneity existed in the ischemic stroke area during the time interval of 24–48 h after stroke onset. As discussed above, other factors could also contribute to it. No matter which explanation is correct, APTW signal inhomogeneity may be also used to differentiate patient with different stroke severity and prognosis.

As for the parameter we used in our study, previous studies indicated the optimal flip angle was 180° (24–26). However, we used a flip angle of 340°. The result of the BSA phantoms showed that a flip angle of 340° was better than a flip angle of 180°. We believed this could be possibly caused by the difference in the B0 and power between our study and the studies we listed. Indeed, the study conducted by Yuki Kanazawa et al. indicated that flip angle of 500 is the optimal flip angle for the APT CEST for the MR scanner (Vantage; Toshiba Medical Systems Corp., Otawara, Japan) they used (27).

There are several limitations for this study. First, we used MTR_asym_ (3.5 ppm) in this study, which was susceptible to the conventional magnetization transfer effect and nuclear Overhauser effect (28–30). Previous studies have shown that these effects are subtle in the case of acute ischemic stroke and therefore MTR_asym_ (3.5 ppm) still could be used as an effective metric to access pH change (31–33). However, the most recent study by Heo et al. revealed that nuclear Overhauser effect would negatively affect APTW signal in ischemic stroke. But it most likely occurs under the circumstance that low saturation power B_1_ (lower than 1–2 μT) is used (34). In this sense, the nuclear Overhauser effect might have some negative effect on the APTW signal since the RF saturation power used in this study was around 1 μT. Plus, previous studies indicated that MTRasym may partially depend on T1 (17, 35, 36), which was not measured in this study. In study conducted by Sun et al. APTW signal was significantly decrease in the ischemic region as the T1 was significantly increased compared with the contralateral normal area. In fact, more precise methods, including model-based approach (37), inverse metric approach (34) and *T*_1_/*T*_2_ time compensated CESTR (38) have been used, which makes APT imaging more sensitive. Second, the sample size was relatively small and further investigations that involve a larger sample size are required. Third, B1 inhomogeneity was not corrected for this study, which may induce error in the CEST imaging and decrease its accuracy. However, it has been indicated that CEST contrast is not very sensitive to B1 inhomogeneity (26, 39). Finally, only one slice was acquired in our study, leaving other part of the stroke area unanalyzed. It would be useful that further study uses a novel three-dimensional APT protocol to assess stroke area which can provide more information to help us fully understand the pH change after ischemic stroke.

In conclusion, quantitative analysis of APTW signal change could be used to assess stroke severity as well as to predict long term clinical outcome for AIS patients. The heterogeneity of APTW signal in the stroke area could also be used as a biomarker to indicate the prognosis. Therefore, APT-weighted imaging could be a promising MRI method for clinical use.

## Author contributions

GL and RW designed the study. GL, CZ, ZS, YC, YS, and XZ performed the research. GL, GX, and ZS analyzed the data. GL wrote the paper. RW and CZ critically revised the manuscript. All the authors approved the final draft.

### Conflict of interest statement

The authors declare that the research was conducted in the absence of any commercial or financial relationships that could be construed as a potential conflict of interest.
